# Corticomedullary mixed tumour resembling a small adrenal gland-involvement of cancer stem cells: case report

**DOI:** 10.1186/s12902-017-0157-7

**Published:** 2017-02-13

**Authors:** Lian Duan, Fang Fang, Wanlei Fu, Zhenqiang Fang, Hui Wang, Shicang Yu, Zili Tang, Zhenqi Liu, Hongting Zheng

**Affiliations:** 1 Department of Endocrinology, Xinqiao Hospital, Third Military Medical University, Xinqiao Street No.183, Shapingba District, Chongqing 400037 China; 2 Department of Endocrinology, Chongqing Hospital of Traditional Chinese Medicine, Chongqing, China; 3 Department of Pathology, Xinqiao Hospital, Third Military Medical University, Chongqing, China; 4 Department of Urology, Xinqiao Hospital, Third Military Medical University, Chongqing, China; 5 Institute of Pathology and Southwest Cancer Center, Southwest Hospital, Third Military Medical University, Chongqing, China; 6 Molecular & Translational Radiation Oncology, Heidelberg Ion Therapy Center, Heidelberg Institute of Radiation Oncology, University of Heidelberg Medical School and National Center for Cancer Diseases, German Cancer Research Center, Heidelberg, Germany; 7 Division of Endocrinology and Metabolism, Department of Medicine, University of Virginia Health System, Charlottesville, VA USA

**Keywords:** Corticomedullary mixed tumour, Tumorigenic mechanism, Cancer stem cell, Case report

## Abstract

**Background:**

Adrenal corticomedullary mixed tumours are very rare. Its mechanism is rarely reported. Here we report the first case of a corticomedullary mixed tumour resembling a “small adrenal gland” with distinct arrangement of the cortical and medullary layers. We further hypothesize regarding the tumorigenic mechanism of this tumour.

**Case presentation:**

A 58-year man had been diagnosed with diabetes and hypertension for 3 years. His 24-h urine vanillylmandelic acid (VMA) levels were slightly elevated. An abnormal circadian cortisol rhythm was noted, and his cortisol levels were not suppressed by dexamethasone. Abdominal computed tomography (CT) revealed a right adrenal gland lesion (diameter, 30 × 38 mm), while an enhanced CT showed enhancement and hypervascularization. The tumour was positive for adrenocorticotropic hormone, chromogranin A (CGA), and steroidogenic factor-1 (SF-1) on the tumour surface. Acetaldehyde dehydrogenase 1(ALDH1), CD44, CD133, Nestin, Nerve growth factor receptor (NGFR), and Sex determining region y-box 9(SOX9) staining were positive. Although administration of medications for diabetes and hypertension was stopped until surgery was performed, the blood sugar level and blood pressure were maintained after surgery.

**Conclusions:**

This is the first report about a possible mechanism by which cancer stem cells induce adrenal corticomedullary tumours.

## Background

The adrenal gland contains a cortex and medulla, which originate from different germ layers. Thus, most primary neoplasms of the adrenal gland arise from either adrenal cortical cells or chromaffin cells of the adrenal medulla, and adrenal tumours generated by more than one cell type are rare. Even if two cell types coexist in the adrenal gland, most often, two separate tumours form. Adrenal cortex and medulla lesions coexisting in the same tumour to form adrenal corticomedullary mixed tumours are rarer. In the approximately 20 cases of adrenal corticomedullary mixed tumours reported to date [1–20], tumour cells were distributed in these tumours in a random and unorganized fashion [11]. Since cases were limited in number and little research on the subject, the tumorigenic mechanism remains unclear. We describe the first case of a corticomedullary mixed tumour resembling a “small adrenal gland” with a distinct arrangement of cortical and medullary layers, and hypothesize that tumour stem cells may contribute to its development.

## Case presentation

**Fig. 1:**
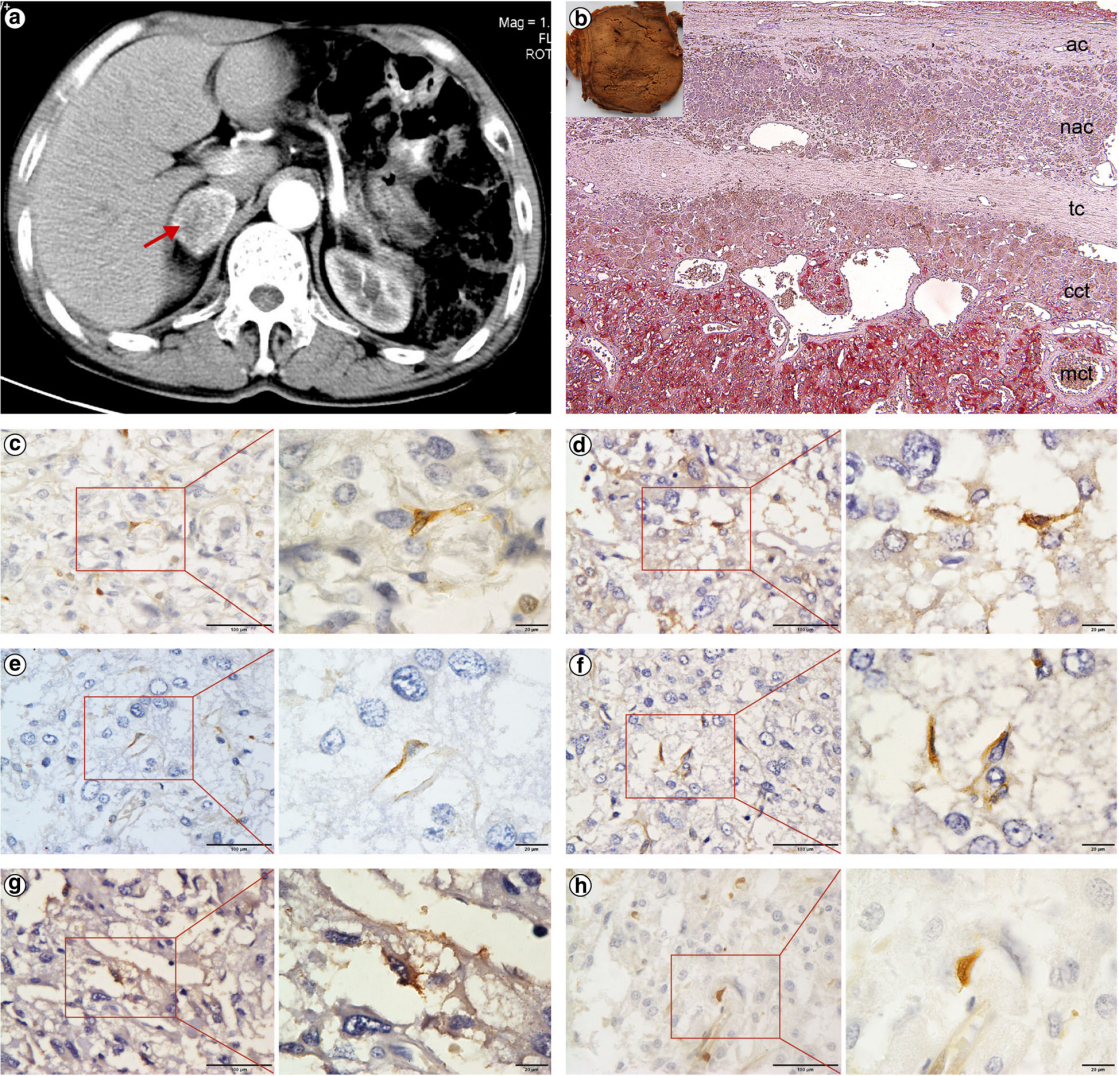
CT and immunohistochemistry of the right adrenal lesion. **a** Abdominal CT image showing a right adrenal lesion (*arrow*). **b** Gross appearance of the resected right adrenal gland, and immunohistochemical double-staining for SF-1 and CGA: the adrenal capsule (ac), residual normal adrenal cortex (nac; stained brown for SF-1), tumour capsule (tc), cortical component of the tumour (cct; stained brown for SF-1), and medullary component of the tumour (mct; stained red for CGA) are visible (×200). **c**-**h** Immunohistochemical staining for the cancer stem cell-specific markers ALDH1 (**c**), CD44 (**d**), CD133 (**e**), Nestin (**f**), NGFR (**g**), and SOX9 (**h**) (×400 and × 1000)

**Fig. 2:**
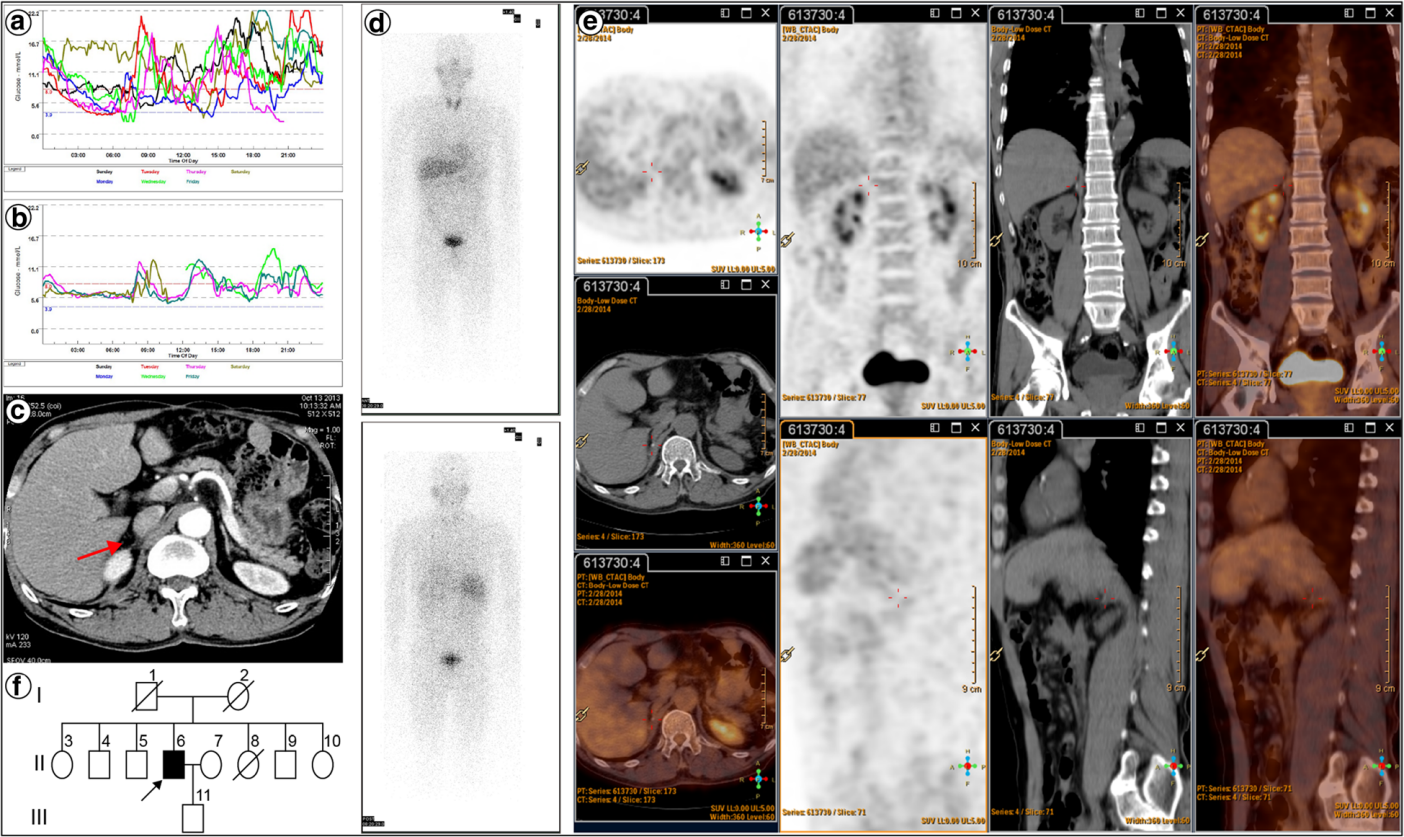
Clinical follow-up datas. **a** A preoperative continuous glucose monitoring graph. **b** A postoperative continuous glucose monitoring graph. **c** A postoperative CT image showing absence of the right adrenal tumour (*arrow*). **d** A metaiodobenzylguanidine scan image. **e** A positron emission tomography-CT image. **f** A pedigree chart

A 58-year-old man with a history of type 2 diabetes and hypertension was admitted in June 2013 because of headache, loss of appetite, constipation, and wide fluctuations in both blood glucose levels (3.3–25.2 mmol/L) and blood pressure (147–180/70–120 mmHg) during the past three months, despite treatment with insulin, metformin, and amlodipine, furthermore, he had undergone subtotal gastrectomy for a stomach ulcer 28 years ago. The patient had no family history of diabetes, hypertension, malignancies, or other related diseases. Physical examination revealed a blood pressure of 119/83 mmHg with no physical signs of moon facies, buffalo hump, truncal obesity, or purple abdominal striae. The CT image revealed a circular, solid, soft tissue mass measuring 30 × 38 mm with a clear boundary in the right side of the adrenal gland. On contrast enhancement, the lesion appeared heterogeneous, with no calcification or necrosis. Enhancement and hypervascularization (Fig. 1a) also were observed (arrow). The radiodensity was 35 hounsfield units (HU) on unenhanced CT and 80 HU on contrast-enhanced CT. Continuous glucose monitoring showed wide fluctuations in blood glucose levels (Fig. 2a), and 24-h urine VMA levels were slightly elevated (Table 1). These clinical manifestations suggested the possibility of pheochromocytoma [21, 22]. Further workup revealed an abnormal circadian cortisol rhythm and cortisol levels that were not suppressed by dexamethasone (either 1 mg overnight or 2 mg every 6 h for 2 days), indicating the possibility of Cushing’s syndrome (Table 1). Other laboratory findings, such as plasma aldosterone, were normal (Table 1). The right adrenal lesion was resected laparoscopically after alpha-adrenergic receptor blockade. Postoperative immunohistochemical staining for SF-1 and CGA confirmed that the tumour was a corticomedullary mixed tumour. In order to analyse the tumorigenic mechanism of corticomedullary mixed tumour with distinct arrangement of the cortical and medullary layers, we stained the resected tissue for various tumour stem cell-specific markers, including ALDH1, CD44, CD133, Nestin, NGFR, and SOX9, and found short spindle-positive cells scattered within the tumour (Fig. 1c-h), suggesting the involvement of tumour stem cells. During 18 months of postoperative follow-up, the patient did not require hypoglycaemic or antihypertensive drugs and had stable blood glucose levels (Fig. 2b) and blood pressure. Additionally, the cortisol rhythm and 24-h urine VMA level returned to normal (Table 1). Abdominal CT confirmed complete resection of the tumour (Fig. 2c), and metaiodobenzylguanidine (Fig. 2d) and positron emission tomography-CT (Fig. 2e) scans revealed no extra-adrenal lesion. Family history screening indicated that this was an isolated case (Fig. 2f).

**Table 1:** The preoperative and postoperative endocrinological evaluation

Parameter	Values		Reference Range
	Preoperative	Postoperative	
ACTH (ng/L)	21.2	39.5	4.8–48.8
Cortisol (nmol/L)	290	392	176.6–579.4(8 am)
	510	196	66–353(4 pm)
	635	146	<100(0 am)
LDDST Cortisol (nmol/L)	232	N/P	<50
HDDST Cortisol (nmol/L)	679	N/P	<145(50% baseline value)
Aldosterone (pg/ml)	42.6	76.3	60–175
UVMA (mg/24h)	16.4	7.60	<13.6
GA (%)	21.64	14.52	11–16
HbA1c (%)	7.7	5.9	4.0–6.0
CPRT (ng/ml) 0 min	0.7	0.79	0.48–0.78
30 min	0.5	5.1	1.3–5.0
60 min	0.6	8.66	1.25–4.6
120 min	1.1	2.17	0.75–3.23
180 min	1.2	0.96	0.37–1.62
FT3 (pmol/L)	2.6	4.2	3.1–6.8
FT4 (pmol/L)	16.6	12.3	11–22
TSH (mIU/L)	1.05	3.96	0.468–4.68
FSH (mIU/ml)	12.29	10.59	0.95–11.95
TEST (nmol/L)	7.73	10.36	1.68–8.11
PROG (ng/ml)	<0.1	0.2	<0.1–0.2
PRL (ng/ml)	8.62	26.14	3.46–19.4
E2 (pg/ml)	23	33	11–44
LH (mIU/ml)	4.51	8.88	1.14–8.75

*ACTH* adrenocorticotrophin, *LDDST* low-dose dexamethasone suppression test, *HDDST* high-dose dexamethasone suppression test, *UVMA* urinary vanillymandelic acid, *GA* glycosylated albumin, *HbA1c* glycosylated haemoglobin; *CPRT* C- peptide release test, *FT3* free triiodothyronine, *FT4* free thyroxine, *TSH* thyroid-stimulating hormone, *FSH* follicle-stimulating hormone, *TEST* testosterone, *PROG* progesterone, *PRL* prolactin, *E2* estradiol, *LH* luteinizing hormone, *N/P* not performed

## Discussion

A corticomedullary mixed tumour is defined as a single adrenal tumour mass containing an intimately admixed population of both adrenal cortical cells and pheochromocytes [1]. Corticomedullary mixed tumours are likely to occur in female [16], and most of them are benign with a favourable prognosis. However, some malignant tumours with a poor prognosis have been reported [16, 20]. Hence, long-term follow-up is needed.

Corticomedullary mixed tumours produce both cortisol and catecholamine, and therefore, signs and symptoms may arise from either tumour component. Hypersecretion of cortical and medullary lesions causes 40% of diabetes cases, 80% of hypertension cases, and 53.33% of Cushing’s syndrome cases [20]. The typical clinical manifestations are easy to identify, however, the lack of specific clinical manifestations in many cases leads to them often being discovered accidentally, and ultimately a pathological diagnosis is required [13]. Cases similar to that of our patient may manifest with subclinical Cushing’s syndrome, including cortisol rhythm abnormalities, but no typical Cushing’s syndrome appearance [11]. Nonspecific symptoms, such as headache and flank pain, can increase the difficulty of diagnosis.

As the adrenal cortex and medulla originate from different germinal layers [23], cortical and medullary tumours usually develop independently, and the simultaneous development of these tumours is rare. Adrenal corticomedullary mixed tumours are rarer. The tumorigenic pathogenesis of such is different from synchronous separate adrenal collision tumours and corticomedullary mixed tumours with random, unorganized cells structure. The question of whether the cortical adenoma and pheochromocytoma components of corticomedullary mixed tumours grow independently, or whether their growth is interrelated, is difficult to resolve [20], therefore, the pathogenesis of corticomedullary mixed tumours remains obscure [16]. The following mechanisms mainly have been proposed: Firstly, pheochromocytomas secreted ectopic corticotropin, which might caused adrenal cortical hyperplasia and/or adrenal cortical adenoma. Moreover, hyper-secreted catecholamines stimulated the anterior pituitary to secrete corticotropin, which may lead to similar adrenal cortical lesions [13]. Additionally, key enzymes of catecholamine synthesis, such as beta hydroxylase, tyrosine hydroxylase, and phenylethanolamine-N-methyltransferase might be regulated by high concentrations of glucocorticoid via the intra-adrenal portal system. This induction leads to increased expression of epinephrine and norepinephrine, thus maintaining a vicious spiral [15]. Besides, researchers speculated that some unknown mechanisms can destroy the interaction of normal cortical-chromaffin cell and result in trophic stimulation of both cells [20]. Finally, genetic mutations in stem cells can lead to adrenal cortical tumours. Cortical tumours have excessive secretion of cortisol, and then leads to induction of the rate limiting enzyme in catecholamine synthesis, i.e. phenylethanolamine-N-methyltransferase, mediated medullary hyperplasia [20].

Concurrent cortical and medullary tumours present as either adrenal collision tumours, in which cortical and medullary tumours develop independently but in close proximity without admixture at the interface [22], or corticomedullary mixed tumours, in which the adrenal cortical and medullary tumour cells develop within the same tumour at random, forming a structurally unorganized tumour without distinct cortical and medullary layers [11]. However, the present tumour differed from those previously described, as the cortical and medullary tumour cells were arranged in distinct layers resembling a “small adrenal gland” (Fig. 1b). The unique features of this tumour indicated that its tumorigenic mechanism might differ from that of other corticomedullary mixed tumours, it was likely derived from a single origin, which was commonly differentiated from tumor stem cell.

## Conclusions

In summary, here we reported the first case of an adrenal corticomedullary mixed tumour with distinct layers of cortical and medullary cells resembling a “small adrenal gland”, which differed from previously reported corticomedullary mixed tumour types. We also hypothesize that tumour stem cells may contribute to the development of this tumour. Our findings provide a new insight into the tumorigenic mechanism of adrenal tumours, and may help future development in adrenal tumour interventions.

## Abbreviations

ALDH1: Aldehyde dehydrogenase 1
CGA: Chromogranin A
CT: Computed tomography
HU: Hounsfield units
NGFR: Nerve growth factor receptor
SF-1: Steroidogenic factor-1
SOX9: Sex determining region y-box 9
VMA: Vanillylmandelic acid
